# In vitro and in vivo evaluation of anti-HER2 antibody conjugates labelled with ^225^Ac

**DOI:** 10.1186/s41181-025-00337-8

**Published:** 2025-04-04

**Authors:** Kateřina Ondrák Fialová, Lukáš Ondrák, Martin Vlk, Ján Kozempel, Kateřina Nováková, Zbyněk Nový, Katarína Hajduová, Marián Hajdúch, Miloš Petřík, Marek Pruszynski, Frank Bruchertseifer, Alfred Morgenstern

**Affiliations:** 1 Department of Nuclear Chemistry, Faculty of Nuclear Sciences and Physical Engineering, Czech Technical University in Prague, Břehová 87/7, 115 19 Prague, Czech Republic; 2 Institute of Organic Chemistry and Biochemistry of the CAS, Flemingovo naměstí 542/2, 16000 Prague, Czech Republic; 3 Institute of Molecular and Translational Medicine, Faculty of Medicine and Dentistry, Palacký University, 779 00 Olomouc, Czech Republic; 4 Institute of Nuclear Chemistry and Technology, Dorodna 16, 03-195 Warsaw, Poland; 5 NOMATEN Centre of Excellence, National Centre for Nuclear Research, Andrzeja Soltana 7, 05-400 Otwock, Poland; 6 European Commission, Joint Research Centre, Karlsruhe, Germany

**Keywords:** HER2, Pertuzumab, Trastuzumab, Actinium-225, Targeted alpha therapy, SKOV-3, MDA-MB-231

## Abstract

**Background:**

Overexpression of human epidermal growth factor receptor type 2 (HER2) occurs in multiple carcinomas. For example, up to 20% of breast cancer cases are classified as HER2 positive (HER2+). Treatment of this condition typically involves immunotherapy using monoclonal antibodies, such as trastuzumab or pertuzumab. The precise targeting of monoclonal antibodies to HER2+ tumour lesions can be used as well in radioimmunotherapy to deliver medical radionuclides exactly to the afflicted area and therefore minimize radiation exposure of healthy tissues. In this study, DOTA conjugates of monoclonal antibodies trastuzumab and pertuzumab were prepared and tested in vitro. One of these, ^225^Ac-DOTA-pertuzumab, was also the subject of an ex vivo biodistribution study with normal as well as HER2+ and HER2- tumour-xenografted mice. This radioconjugate has not been previously described.

**Results:**

Three DOTA-conjugates of HER2 targeting monoclonal antibodies, one of trastuzumab and two of pertuzumab, were prepared and radiolabelled with ^225^Ac in different molar ratios. This procedure led to an optimisation of the preparation and radiolabelling process. The radioconjugates were shown to be highly stable in vitro in both fetal bovine serum and phosphate buffered saline under room temperature and decreased temperature for 10 days. In vitro cell studies with HER2-overexpressing cell-line (SKOV-3) and low HER2-expressing cell line (MDA-MB-231) proved that radioconjugates of both antibodies have high binding specificity and affinity towards HER2 receptors. These findings were confirmed for a novel radioconjugate ^225^Ac-DOTA-pertuzumab in an ex vivo biodistribution study, where uptake in HER2+ tumour was 50 ± 14% ID/g and HER2- tumour showed uptake comparable with healthy tissues (max. 5.0 ± 1.7% ID/g). The high uptake observed in the spleen can be attributed to the elimination of the antibody, as well as the use of an immunedeficient mouse strain (SCID).

**Conclusions:**

During this study, the optimization of preparation and radiolabelling of HER2 targeting antibodies with ^225^Ac was accomplished. Furthermore, the radioconjugate ^225^Ac-DOTA-pertuzumab was prepared and evaluated for the first time. The radioconjugates of both tested antibodies demonstrated excellent qualities in terms of stability and HER2 receptor affinity. Initial ex vivo studies indicated that especially the radioconjugate ^225^Ac-DOTA-pertuzumab is a very promising candidate for further more detailed in vivo studies.

**Supplementary Information:**

The online version contains supplementary material available at 10.1186/s41181-025-00337-8.

## Background

The human epidermal growth factor receptor type 2 (HER2) is a transmembrane glycoprotein of approximately 185 kDa, encoded by HER2 protooncogene on chromosome 17 (Mitri et al. 2012). It belongs to the family of epidermal growth factor receptors together with HER1, HER3 and HER4. All HER receptors are present on the cell surface in the form of monomers. Upon binding of specific ligands the homo- or heterodimerization of receptors is initiated resulting in the activation of signalling pathways to cell growth, survival, proliferation, differentiation and angiogenesis. Receptor HER2 is the only receptor whose dimerization is ligand independent. Hence, it is a favourable partner for dimerization and as such it is the strongest stimulator of cell proliferation pathways (Iqbal and Iqbal 2014; Rubin and Yarden 2001).

At normal physiological levels, it supports normal cell growth. When overexpressed, the proliferative activity is increased leading to malignant growth (Rubin and Yarden 2001; Neve et al. 2001). Overexpression of HER2 receptor is observed in up to 20% of breast cancer cases. It is also observed in high abundance in gastric, ovarian, endometrial, cervical, colorectal and head and neck cancers. Due to the agressiveness of diseases it causes, HER2 positivity is a crucial factor in evaluation of cancer treatment prognosis (Angelis and Okines 2024; Stanowicka-Grada et al. 2023; Mitri et al. 2012; Iqbal and Iqbal 2014; Wang and Xu 2019).

Immunotherapy with monoclonal antibodies combined with chemotherapy is usually the preferred approach in the treatment of HER2+ carcinomas. Trastuzumab, the first monoclonal antibody approved for the treatment of HER2+ breast cancer and gastric adenocarcinoma, is now routinely used even in the early stages of the disease. In 2012 the FDA approved another monoclonal antibody, pertuzumab, which is now used in combination with trastuzumab and chemotherapy for metastatic and even early-stage of HER2+ breast cancer (Angelis et al. 2023; Kreutzfeldt et al. 2020; SmPC Herceptin; SmPC Perjeta).

The main issue with the use of these monoclonal antibodies is related to a relatively fast gained resistance to treatment (Melo et al. 2016). Therefore, further research of alternative treatment is crucial. Besides monoclonal antibodies, inhibitors of tyrosine kinases, such as Lapatinib, are used. Another option is the use of monoclonal antibody conjugates with cytotoxic drugs (antibody–drug conjugates, ADC). Here monoclonal antibodies serve as a targeting molecule carrying chemotherapeutics (Angelis and Okines 2024; Kreutzfeldt et al. 2020).

**Fig. 1:**
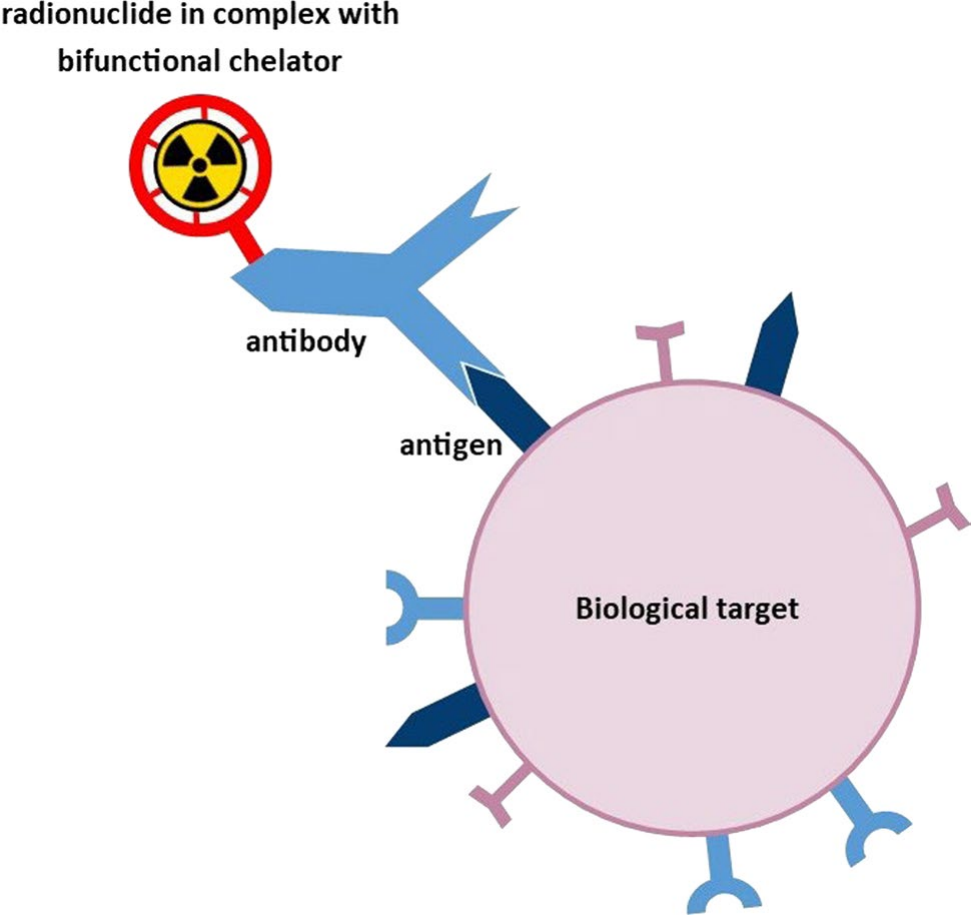
Concept of a radiopharmaceutical for radioimmunodiagnostics or therapy

A comparable approach can be used also in nuclear medicine in radioimmunodiagnostics and therapy. In diagnostics, utilising SPECT or PET the precise targeting enables to get a maximal signal/noise ratio and gain exact image of disease extent. In therapy, the radionuclide decay and absorption of ionizing radiation happens in the afflicted area (the target) and the therapeutic index is maximized (Larson et al. 2015; Rondon et al. 2021). A concept of such a radiopharmaceutical is depicted in Fig. 1 on an example of a radiometal conjugated via bifunctional chelator to the antibody.

Thanks to the well-known pharmacokinetics and pharmacodynamics, both trastuzumab and pertuzumab have been tested for use in radioimmunodiagnostics or radioimmunotherapy with various radionuclides and bifunctional chelators connecting those two together.

**Fig. 2:**
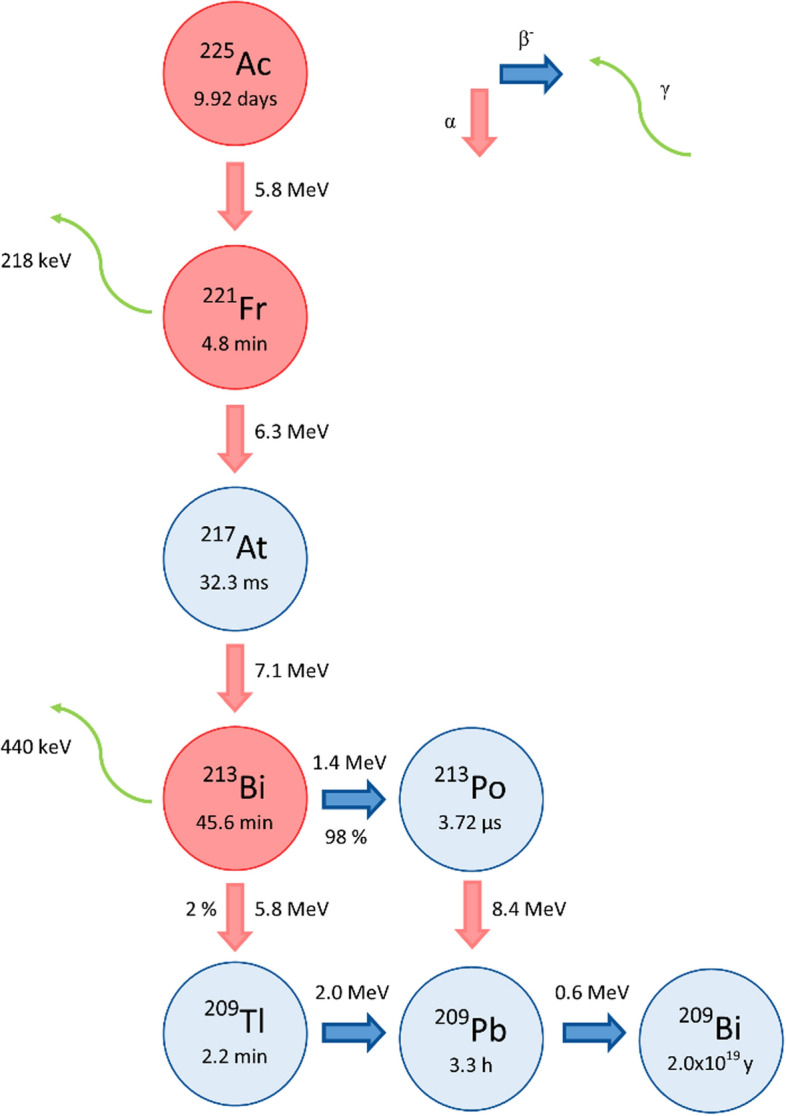
^225^Ac decay scheme with clinically significant gamma emissions

In relation to the biological half-life of trastuzumab and pertuzumab, which is 28.5 and 18 days, respectively (SmPC Herceptin, SmPC Perjeta), the diagnostical radionuclide of choice is very often ^89^Zr (*T*_1/2_ = 3.3 d) or ^111^In (*T*_1/2_ = 2.8 d) (Kang et al. 2022; Lub-de Hooge et al. 2004; Dijkers et al. 2009; O'Donoghue et al. 2018; Perik et al. 2006; Bensh et al. 2018; Ulaner et al. 2018). For therapy, well established *β*^−^ emitters ^131^I (*T*_1/2_ = 8 d) or ^177^Lu (*T*_1/2_ = 6.7 d) were tested (Puttemans et al. 2020; Rasaneh et al. 2010b; Guleria et al. 2020; Narwadkar et al. 2024; Menon et al. 2022). Nevertheless, the concept of precise targeting in radioimmunotherapy might be especially beneficial with use of *α* emitters. This is the domain of targeted alpha therapy (TAT), a very fast developing branch of nuclear medicine. The primary advantage of *α* emitters is their high linear energy transfer (LET) of 50–100 keV/μm and their short range of 40–80 μm in tissue comparing to *β*^−^ emitters. That leads to highly localised enormous energy deposition (Morgenstern et al. 2018; Hooijman et al. 2024). The key radionuclide of TAT is ^225^Ac with a half-life of 9.92 days. Every complete decay of ^225^Ac leads to the emission of 4 *α* particles with energy range 5.8–8.4 MeV and 2 *β*^*−*^ particles of energy 0.6–2.0 MeV (see Fig. 2). This results in an energy output of approximately 28 MeV per complete decay, which is significantly higher than the maximum energy of 497.4 keV produced by the decay of ^177^Lu. Besides ^225^Ac, there are other important radionuclides in its decay chain, including ^218^Fr and ^213^Bi, which emit γ radiation of 218 and 440 keV, respectively, suitable for detection (Hooijman et al. 2024). Acitinium-225 has already been successfully implemented in clinical studies with peptides in patients with prostate, glioma or neuroendocrine cancers (Kratochwil et al. 2014, 2016, 2017; Krolicki et al. 2016). Furthermore, it has been included in a few studies with trastuzumab (Kondo et al. 2023; Borchardt et al. 2003) and a HER2 targeting nanobody (Pruszynski et al. 2018; Rodak et al. 2022). Among other *α* emitters considered for use in radioimmunotherapy of HER2+ diseases there are ^227^Th (*T*_1/2_ = 18.7 d), ^212^Pb (*T*_1/2_ = 10.6 h), ^211^At (*T*_1/2_ = 7.2 h) or ^213^Bi (*T*_1/2_ = 45.6 min) (Abbas et al. 2011; Milenic et al. 2005; Li et al. 2021; Dekempeneer et al. 2020; Palm et al. 2007).

The significant benefit of *α* emitters can also become a huge drawback as the release of the radionuclide from its original form can result in substantial irradiation of healthy tissues. In the context of cascade decaying radionuclides or so-called in vivo generators, such as ^225^Ac, the issue of the recoil effect must be taken into consideration, as the recoil of the daughter radionuclide from its original form can also rise the radiation burden (Sakmár et al. 2024). The stability of prepared radioconjugate, as well as the high degree of internalization, are then pivotal parameters for new radiopharmaceuticals in TAT (Hooijman et al. 2024; Scheinberg and McDevitt 2011).

In the majority of studies focusing on radioimmunodiagnostics or therapy of HER2+ diseases, trastuzumab is the monoclonal antibody of choice, leaving pertuzumab aside. To date, pertuzumab has been used in several studies with ^89^Zr (Marquez et al. 2014; Sharma et al. 2018; Lee et al. 2019) or ^177^Lu (Menon et al. 2022), and to the best of our knowledge, no study with ^225^Ac has been reported, yet. However, due to its comparatively shorter biological half-life, use of pertuzumab can be more convenient in terms of reducing the radiation burden on healthy tissues during the distribution phase. That is why it is the key antibody in the present study.

Here, we present the synthesis and characterization of trastuzumab and pertuzumab conjugates with DOTA chelator. The prepared conjugates were labelled with ^225^Ac and then tested for stability, binding specificity and affinity towards HER2 receptor in vitro. One of the conjugates, ^225^Ac-DOTA-pertuzumab, was also tested in an ex vivo biodistribution study in normal and tumour-xenografted mice.

## Methods

### Chemicals and radionuclides

All used chemicals were Ph. Eur. grade purchased from Merck, Germany unless otherwise stated. Acids were of ultrapure grade (VWR Chemicals, USA). Monoclonal antibodies trastuzumab and pertuzumab for synthesis were purchased from MedChemExpress, USA. The bifunctional chelator 1,4,7,10-tetraazacyclododecane-1,4,7,10-tetraacetic acid mono-*N*-hydroxysuccinimide ester (DOTA-NHS) was purchased from CheMatech, France. Phosphate buffered saline (PBS) was prepared from tablets (PanReac AppliChem, Germany). Ultrapure water was prepared on Direct Q3 (Millipore, USA).

Actinium-225 was produced at the Joint Research Centre (Karlsruhe, Germany) via radiochemical extraction from a ^229^Th source. The activity of ^225^Ac was always measured after reaching equilibrium with its daughter products.

### Synthesis of conjugates

Trastuzumab or pertuzumab (30 nmol) was mixed in 1 M borate buffer (pH 8) with 20-fold or 40-fold molar excess of bifunctional chelator DOTA-NHS. The reaction mixture was shaken for 4 h at 37 °C and then stored in the fridge overnight.

The product was purified by multiple rinses on Vivaspin 500 concentrators with 100 kDa cutoff filters (Sartorius, Germany). The number of conjugated chelator molecules were assessed by matrix-assisted laser desorption/ionization time of flight mass spectrometry (UltrafleXtreme™ MALDI-TOF–MS, Bruker Daltonics, Germany). The concentration of conjugate was determined using UV–Vis spectrometer CARY 100 (Varian, USA).

### Radiolabelling and in vitro stability tests

The prepared conjugates were radiolabelled with ^225^Ac in various conjugate:radionuclide ratios in 0.02 M ammonium acetate (pH 6) at 37 °C for 2 h. The activity of samples was verified using ionizing chamber Curiementor 2 (PTW, Germany).

Radiolabelling yields were determined by thin layer chromatography (TLC) on ITLC-SG (Agilent Technologies, USA) using 0.05 M natrium citrate (pH 5.5). The radiochromatograms were measured on TLC scanner AR 2000 (Bioscan Inc., USA) after 24 h. The radiolabelled conjugate was retained at the origin (*R*_f_ = 0.0), free ^225^Ac moved with the front (*R*_f_ = 0.8–1.0) and the complex of free DOTA with ^225^Ac remained at *R*_f_ = 0.6–0.8.

For other tests the radiolabelled conjugate was purified on size-exclusion column PD10 (Cytiva, USA) preconditioned with fetal bovine serum (FBS) and washed with large amount of PBS.

The stability of purified radioconjugates of both antibodies were tested in PBS and FBS at both room and decreased temperature (4 °C) for 10 days. The radiochemical purity of radioconjugates was determined in time intervals by TLC as mentioned above.

### Cell lines

Cell lines SKOV-3 (HER2-overexpressing) and MDA-MB-231 (low HER2-expressing) were obtained from American Type Culture Collection. SKOV-3 cells were cultivated in McCoy´s 5A medium and MDA-MB-231 cells in DMEM medium (both Biowest, France). Both media were supplemented by 10% of heat-inactivated fetal bovine serum (Biowest, France), L-glutamine, and streptomycin (100 μl/ml) and penicillin (100 UI/ml) (ThermoFisher Scientific, USA). Cells were cultivated in 5% CO_2_ humidified atmosphere at 37 °C. For rinsing, sterile solution of PBS (Biowest, France) was used. Before experiment the cells were detached using trypsin 1x (Biowest, France).

### Binding specificity assay

The HER2 binding specificity was tested using both SKOV-3 and MDA-MB-231 cells. The cells (8 × 10^4^ per well) were adhered overnight in 24-well plates (12 wells for SKOV-3, 12 wells for MDA-MB-231). The cells were then rinsed with PBS and the radioconjugate (30 nM) was added with fresh medium followed by incubation for 2 h at 4 °C with or without the 100-fold molar excess of cold antibodies trastuzumab or pertuzumab. Each sample was done in triplicates. After incubation the cells were rinsed twice with PBS. Medium and both rinses represented unbound activity of radioconjugate. The cells were lysed twice with 1 M NaOH for 10 min at 37 °C. The lysate represented bound activity. All samples were measured after 24 h in a well-type detector NaI(Tl) (Tesla, Czechoslovakia) or in an automatic γ-counter Wizard 2480 (PerkinElmer, USA).

### Binding affinity assay

The saturation assay was used to evaluate HER2 binding affinity of prepared radioconjugates. SKOV-3 cells (8 × 10^4^ per well) were adhered overnight in two 24-well plates. The cells were then washed with cold PBS and incubated with increasing concentrations (0.046–100 nM) of radioconjugate for 2 h at 4 °C. The first plate served for total binding (specific and non-specific), the second plate contained also 100-fold molar excess of cold antibodies trastuzumab or pertuzumab to distinguish nonspecific binding. Each sample was done in triplicates. After incubation the same procedure as in case of binding specificity assay was followed. By non-linear regression of the data for specific binding the dissociation constant of the conjugate *K*_D_ and the concentration of the radioconjugate necessary for saturation of receptors *B*_max_ were calculated in Origin 2023b.

### Ex vivo biodistribution study

All animal experiments were done at The Institute of Molecular and Translational Medicine in Olomouc following the regulations and guidelines of the Czech Animal Protection Act (No.246/1992) and with the approval of Ministry of Education, Youth and Sports of the Czech Republic and the Institutional Animal Welfare Committee of the Faculty of Medicine and Dentistry of Palacký University in Olomouc.

Animal studies were performed only in case of ^225^Ac-DOTA-pertuzumab radioconjugate. The ex vivo biodistribution studies were done with both normal female BALB/c mice (ENVIGO, Indianapolis, IN, USA) and tumour-xenografted SCID mice (ENVIGO, Indianapolis, IN, USA). The SCID mice were inoculated in the right side of the chest with 2 × 10^6^ SKOV-3 cells in 50% Matrigel (Corning, USA) 53 days before experiment and in the left side of the chest with the same number of MDA-MB-231 cells 21 days before experiment. The mean tumour volume at the beginning of the experiment was 600 mm^3^. Five groups of normal or tumour-xenografted mice (*n* = 4) were injected retro-orbitally with 10.0 kBq/animal of ^225^Ac-DOTA-pertuzumab (sample ^225^Ac-PD2). The groups were euthanized in time intervals 1 h, 1, 2, 3, 7 days p.i. followed by the collection of blood, organs (spleen, pancreas, stomach, intestine, kidneys, liver, heart, lungs, muscle, bone) and tumours that were weighted, and their activity was measured after 24 h in an automatic γ-counter Wizard 2480 (PerkinElmer, USA) together with standards of injected ^225^Ac-DOTA-pertuzumab. The results were expressed as the percentage of injected dose per gram of respective tissue (% ID/g).

## Results

### Synthesis, radiolabelling and in vitro stability

Both trastuzumab and pertuzumab were successfully conjugated with DOTA-NHS bifunctional chelator via peptide bond with *ε*-amino groups of lysines in the antibody structure. Three conjugates were prepared, and MALDI-TOF–MS confirmed the number of conjugated DOTA molecules. It was shown that in order to achieve the same amount of conjugated DOTA molecules for both antibodies, double molar excess of bifunctional chelator is necessary in case of pertuzumab (see Table 1). All three conjugates were radiolabelled with ^225^Ac in a 1:2000 molar ratio of radionuclide:conjugate to obtain preliminary data. The sufficient radiolabelling yield was achieved only in case of those conjugates containing 6 DOTA molecules (TD1 and PD2). The results are summarized in Table 1. The mass spectra of the conjugates, together with spectra of antibodies, are depicted in supplementary material (see Fig. S1-S5), along with the model radiochromatograms from the preliminary labelling experiment (see Fig. S6-S8).

**Table 1:** Characteristics of prepared conjugates: molar excess of bifunctional chelator, number of conjugated DOTA molecules (*N*) and yield of radiolabelling with ^225^Ac in ration radionuclide:conjugate 1:2000

ID	Antibody	DOTA-NHS molar excess [-]	*N* [-]	*Y*_Ac-225_ [%]
TD1	Trastuzumab	20	6.0	75.1
PD1	Pertuzumab	20	1.4	27.9
PD2	Pertuzumab	40	6.0	81.5

**Fig. 3:**
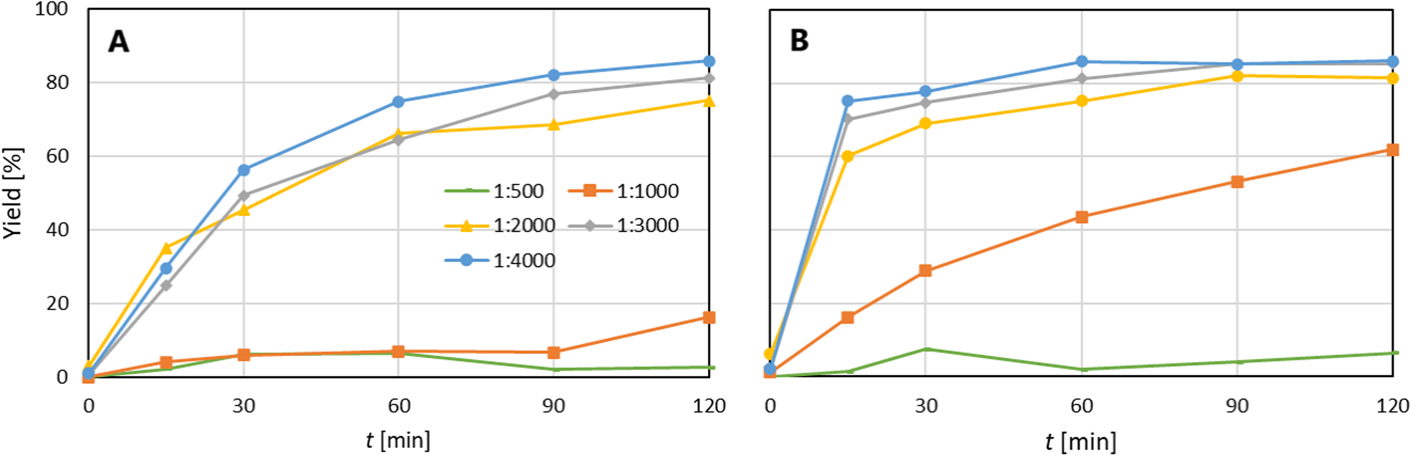
Dependence of yield of radiolabelling of TD1 and PD2 with ^225^Ac on time for various radionuclide:conjugate ratios (0.2 M ammonium acetate, pH 6, 37 °C); **A**: ^225^Ac-TD1, **B**: ^225^Ac-PD2

The optimization of the radiolabelling procedure was conducted for conjugates TD1 and PD2. The results are depicted in Fig. 3. It can be seen that optimal radiolabelling yields is achieved for a 1:2000 molar ratio of radionuclide:conjugate with radiolabelling yields over 75 and 80% for TD1 and PD2, respectively. It is evident that any other increase in conjugate amount leads to minimal rise in radiolabelling yields and significant decrease of specific activity.

For further studies prepared radioconjugates, ^225^Ac-TD1 and ^225^Ac-PD2, were purified on size-exclusion column. This method ensured the radiochemical purity over 99% and the specific activity of radioconjugates over 1.21 MBq/mg for ^225^Ac-TD1 and 1.26 MBq/mg for ^225^Ac-PD2.

**Fig. 4:**
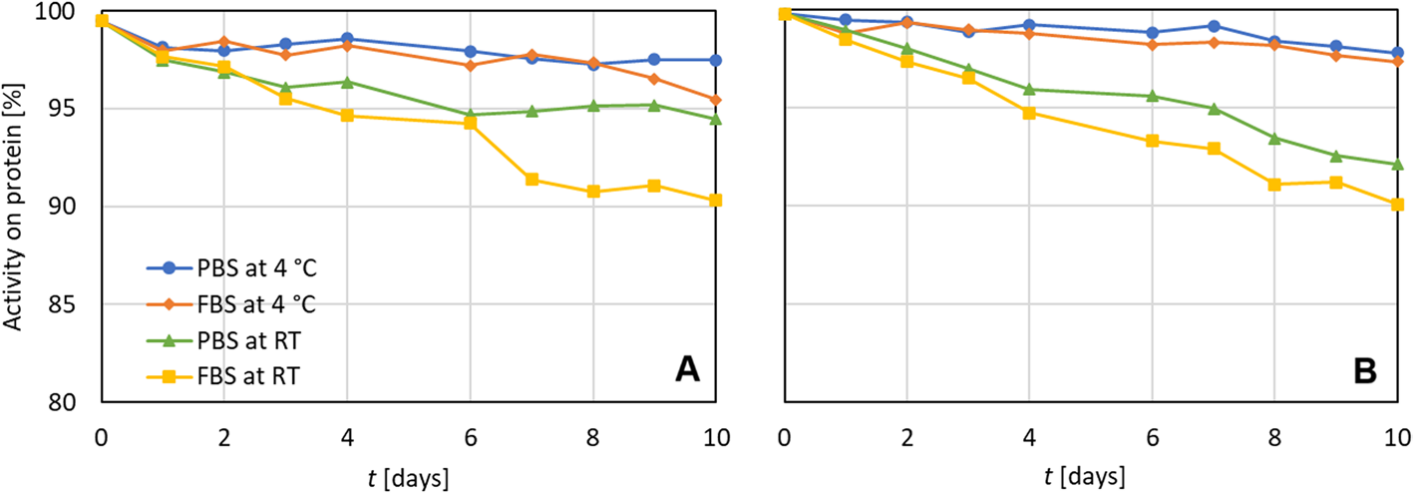
Stability of purified radioconjugates in PBS and FBS at 4 °C and at room temperature (RT); **A**: ^225^Ac-TD1, **B**: ^225^Ac-PD2

The in vitro stability tests of the purified radioconjugates were then assessed. The results are summarized in Fig. 4. It was confirmed that the radiochemical purity (activity on protein) of both conjugates stored in PBS and FBS at decreased temperature (4 °C) did not decrease below 95% for 10 days. Samples stored at room temperature also showed good stability as the radiochemical purity did not decrease below 90%.

### Binding specificity and affinity

**Fig. 5:**
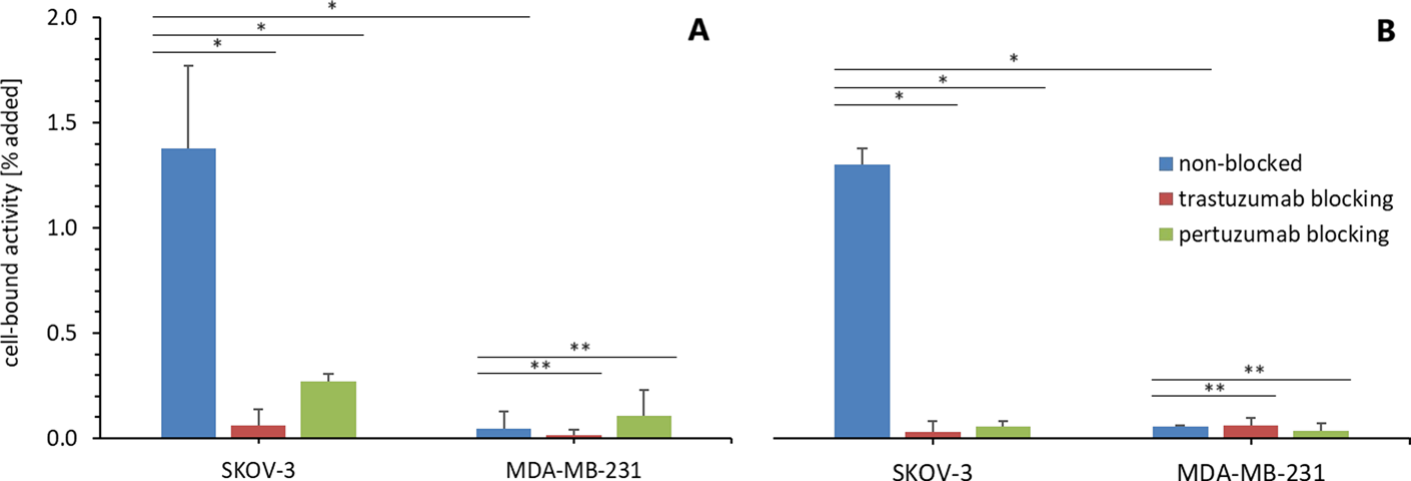
HER2 binding specificity of radioconjugates to SKOV-3 and MDA-MB-231 cell lines non-blocked or trastuzumab/pertuzumab blocked; mean ± SD (*n* = 3); *P < 0.05; **P > 0.1; **A**: ^225^Ac-TD1, **B**: ^225^Ac-PD2

Both radioconjugates, ^225^Ac-TD1 and ^225^Ac-PD2, demonstrated binding specificity towards HER2 receptors on SKOV-3 cells in comparable range (TD1: 1.4 ± 0.4% and PD2: 1.31 ± 0,08% of total activity) as it is shown in Fig. 5. There is no significant binding of radioconjugates in case of SKOV-3 cells blocked by 100-fold molar excess of both cold antibodies in comparison with non-blocked cells (TD1: trastuzumab *P* < 0.05, pertuzumab *P* < 0.005; PD2: trastuzumab *P* < 0.00002, pertuzumab *P* < 0.00002). Also, the binding to MDA-MB-231 is negligible in comparison with binding to SKOV-3 cell (TD1: *P* < 0.005; PD2: *P* < 0.00001) and that does not change even for MDA-MB-231 cells blocked with 100-fold molar excess of either one of cold antibodies (*P* > 0.1).

**Fig. 6:**
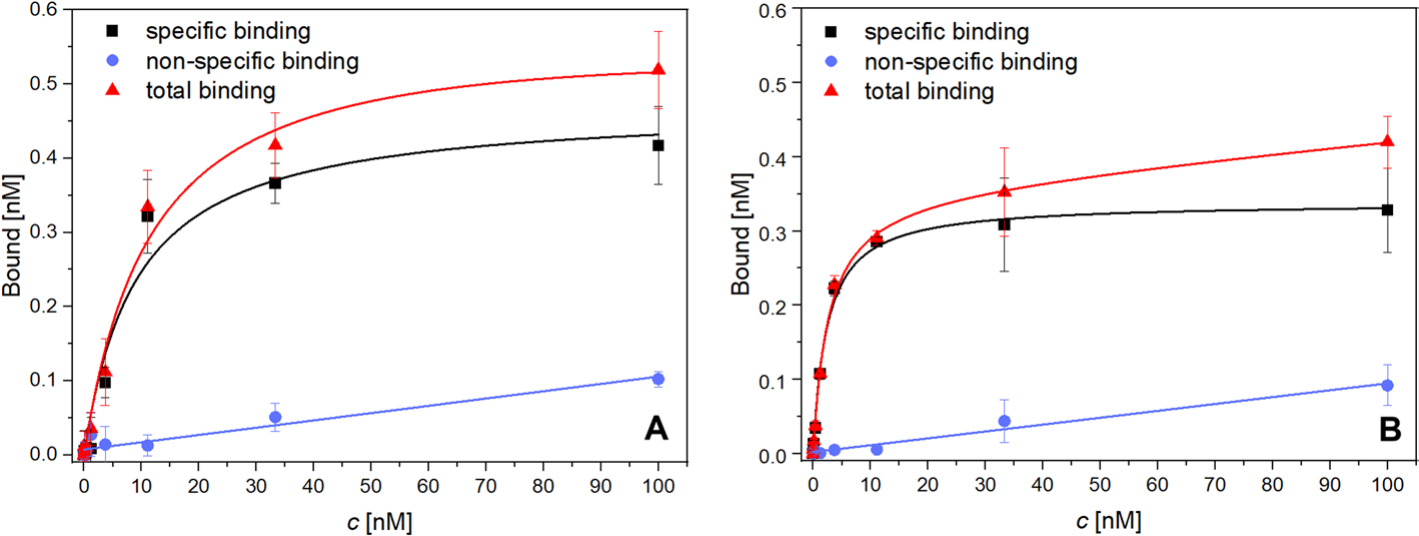
Results of the saturation binding assay of radioconjugates on SKOV-3 cell line, specific, non-specific and total binding; *n* = 3 for each data points; **A**: ^225^Ac-TD1, **B**: ^225^Ac-PD2

From saturation assay with SKOV-3 cell line, the dissociation constant *K*_D_ of the conjugates and the concentration of the radioconjugate necessary for saturation of receptors *B*_max_ were determined. The values are summarized in Table 2 and the saturation curves for both radioconjugates are depicted in Fig. 6.

**Table 2:** Dissociation constants *K*_D_ and the saturation concentrations *B*_max_ of prepared radioconjugates for HER2 receptors (SKOV-3 cell line)

ID	*K*_D_ [nM]	*B*_max_ [nM]
^225^Ac-TD1	9 ± 3	0.48 ± 0.04
^225^Ac-PD2	2.4 ± 0.2	0.339 ± 0.008

### Ex vivo biodistribution study

**Fig. 7:**
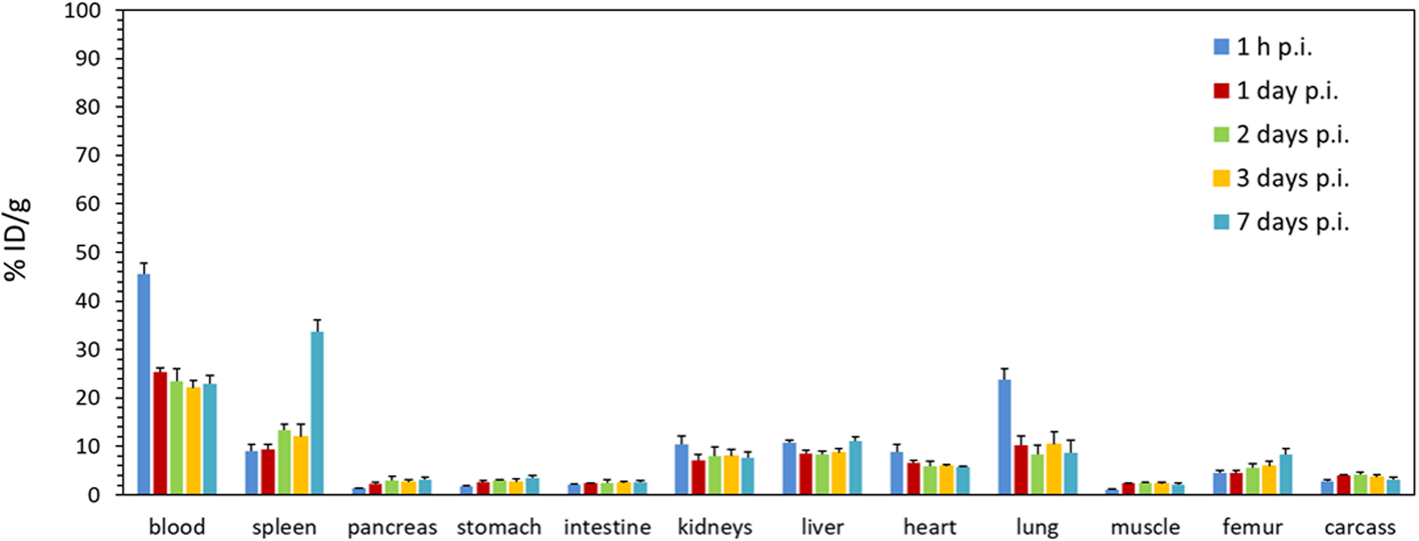
Ex vivo biodistribution of.^225^Ac-DOTA-pertuzumab (10 kBq/animal) in normal female BALB/c mice; mean ± SD (*n* = 4)

The biodistribution of ^225^Ac-DOTA-pertuzumab (^225^Ac-PD2) in normal mice over time is depicted in Fig. 7 and Table 3. Table 4 shows mass of individual organs. The decrease of activity in blood between intervals 1 h and 1 day p.i. is evident. After that it remained around the average value of 23 ± 2% ID/g. Low uptake was observed in case of muscle, pancreas, stomach and intestine (below 3% ID/g). Slightly higher uptake in bones, from average 5.9 ± 1.2% ID/g to 8.4 ± 1.3% ID/g, between 3 and 7 days p.i. can be seen. The uptake up to 10% was observed in highly perfused organs such as lung, heart or kidneys and in liver as the elimination organ of the radioconjugate. The increasing uptake in spleen can be seen between 3 and 7 days p.i from average 12 ± 2% ID/g up to 34 ± 8% ID/g. This correlates with the metabolism of the radioconjugate and the observed spleen atrophy.

**Table 3:** Ex vivo biodistribution of ^225^Ac-DOTA-pertuzumab (10 kBq/animal) in normal female BALB/c mice; mean ± SD (*n* = 4)

Tissue/Organ	% ID/g				
	1 h	1 day	2 days	3 days	7 days
Blood	46 ± 2	25.4 ± 0.8	23 ± 3	22.1 ± 1.6	23 ± 3
Spleen	9.1 ± 1.3	9.3 ± 1.1	13.3 ± 1.4	12 ± 2	34 ± 8
Pancreas	1.3 ± 0.1	2.3 ± 0.3	3.0 ± 0.9	2.6 ± 0.5	3.2 ± 0.4
Stomach	1.80 ± 0.12	2.7 ± 0.2	2.9 ± 0.2	2.7 ± 0.6	3.5 ± 0.6
Intestine	2.17 ± 0.14	2.39 ± 0.11	2.5 ± 0.6	2.4 ± 0.5	2.6 ± 0.4
Kidneys	10.4 ± 1.8	7.1 ± 1.3	8 ± 2	8.1 ± 1.3	7.6 ± 0.6
Liver	10.7 ± 0.6	8.6 ± 0.6	8.2 ± 0.7	8.7 ± 0.8	11 ± 1
Heart	8.9 ± 1.6	6.6 ± 0.6	5.8 ± 1.2	6.0 ± 0.3	5.7 ± 0.6
Lung	24 ± 2	10 ± 2	8.3 ± 1.9	10 ± 2.6	9 ± 1
Muscle	1.0 ± 0.2	2.36 ± 0.15	2.4 ± 0.2	2.2 ± 0.4	2.11 ± 0.17
Femur	4.5 ± 0.6	4.5 ± 0.6	5.6 ± 0.9	5.9 ± 1.2	8.4 ± 1.3
Carcass	2.8 ± 0.4	4.08 ± 0.14	4.1 ± 0.5	3.7 ± 0.5	3.1 ± 0.2

**Table 4:** Average mass of tissues and organs gathered at indicated time points during e*x vivo* biodistribution study of ^225^Ac-DOTA-pertuzumab in normal female BALB/c mice; mean ± SD (*n* = 4)

Tissue/organ	*m* [mg]				
	1 h	1 day	2 days	3 days	7 days
Blood	530 ± 60	460 ± 80	430 ± 100	400 ± 120	450 ± 30
Spleen	81 ± 8	62 ± 5	54 ± 5	49 ± 4	20 ± 7
Pancreas	143 ± 16	114 ± 6	200 ± 120	160 ± 14	130 ± 30
Stomach	159 ± 13	157 ± 8	139 ± 18	150 ± 30	120 ± 30
Intestine	1990 ± 160	1990 ± 150	1690 ± 200	2000 ± 200	1790 ± 150
Kidneys	249 ± 16	236 ± 13	250 ± 40	243 ± 18	240 ± 60
Liver	960 ± 8%	890 ± 50	870 ± 100	930 ± 80	730 ± 80
Heart	90 ± 9	95 ± 8	90 ± 20	92 ± 5	64 ± 11
Lung	241 ± 11	180 ± 40	140 ± 20	200 ± 70	111 ± 15
Muscle	270 ± 40	260 ± 50	240 ± 20	240 ± 40	160 ± 30
Femur	21 ± 6	24 ± 4	24 ± 4	26 ± 9	21 ± 6
Carcass	13,810 ± 630	13,170 ± 570	13,000 ± 1500	13,000 ± 1000	12,290 ± 840

**Fig. 8:**
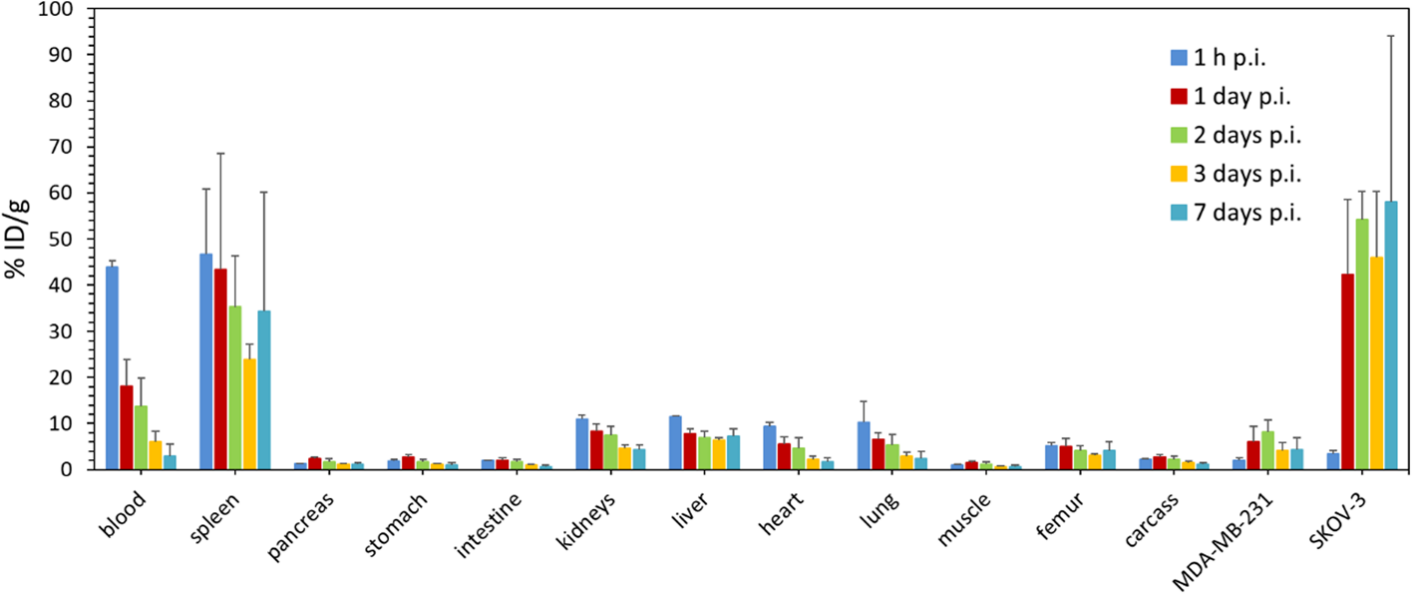
Ex vivo biodistribution of.^225^Ac-DOTA-pertuzumab (10 kBq/animal) in female SCID mice bearing SKOV-3 (HER2 overexpressing) and MDA-MB-231 (HER2 low expressing) tumour xenografts; mean ± SD (*n* = 4)

In Fig. 8 and Table 5, the biodistribution of ^225^Ac-PD2 in tumour-xenografted mice over time can be seen. Table 6 shows average mass of individual organs. The exponential decrease of activity in blood from 43.9 ± 1.3%ID/g to 2.8 ± 2.7% ID/g can be seen. Most organs and tissues showed similar uptake as in case of normal mice (below 2.5% ID/g). The observed decrease in uptake in highly blood perfused organs correlates with the decrease of activity in blood over time. No increase of uptake was observed (average value of 4.3 ± 1.5% ID/g) in bones. However, there was a significant increase of uptake in spleen (average value of 37 ± 20% ID/g) compared to the normal mice. The high SD probably comes from both intra and intergroup scatter in spleen size.

**Table 5:** Ex vivo biodistribution of ^225^Ac-DOTA-pertuzumab (10 kBq/animal) in female SCID mice bearing SKOV-3 (HER2 overexpressing) and MDA-MB-231 (HER2 low expressing) tumour xenografts; mean ± SD (*n* = 4)

Tissue/organ	% ID/g				
	1 h	1 day	2 days	3 days	7 days
Blood	43.9 ± 1.3	18 ± 6	14 ± 6	6 ± 2	2.8 ± 2.7
Spleen	47 ± 14	43 ± 25	35 ± 11	24 ± 3	34 ± 26
Pancreas	1.24 ± 0.12	2.3 ± 0.4	1.7 ± 0.6	1.15 ± 0.17	1.2 ± 0.4
Stomach	1.8 ± 0.3	2.7 ± 0.5	1.7 ± 0.6	1.08 ± 0.17	1.0 ± 0.4
Intestine	2.0 ± 0.1	2.0 ± 0.5	1.6 ± 0.6	0.90 ± 0.18	0.7 ± 0.3
Kidneys	11.0 ± 0.9	8.3 ± 1.6	7.5 ± 1.9	4.6 ± 0.8	4.2 ± 1.1
Liver	11.4 ± 0.2	7.8 ± 1.1	7.0 ± 1.4	6.4 ± 0.6	7.3 ± 1.4
Heart	9 ± 1	5.5 ± 1.6	4 ± 2	2.1 ± 0.8	2 ± 1
Lung	10 ± 5	6.5 ± 1.4	5 ± 2	2.9 ± 0.9	2.4 ± 1.5
Muscle	0.9 ± 0.2	1.4 ± 0.5	1.2 ± 0.4	0.6 ± 0.1	0.7 ± 0.3
Femur	5.2 ± 0.7	5.0 ± 1.7	4 ± 1	3.1 ± 0.4	4 ± 2
Carcass	2.2 ± 0.2	2.7 ± 0.5	2.2 ± 0.7	1.5 ± 0.3	1.2 ± 0.4
MDA-MB-231 tumour	2.1 ± 0.4	6 ± 3	8 ± 3	4.2 ± 1.6	4 ± 3
SKOV-3 tumour	3.5 ± 0.6	42 ± 16	54 ± 6	46 ± 14	58 ± 36

**Table 6:** Average mass of tissues and organs gathered at indicated time points during e*x vivo* biodistribution study of ^225^Ac-DOTA-pertuzumab in tumour-xenografted female SCID mice; mean ± SD (*n* = 4)

Tissue/Organ	*m* [mg]				
	1 h	1 day	2 days	3 days	7 days
Blood	700 ± 120	800 ± 60	660 ± 200	700 ± 100	490 ± 70
Spleen	44 ± 16	32 ± 18	22 ± 8	36 ± 8	31 ± 23
Pancreas	154 ± 8	140 ± 40	170 ± 70	140 ± 40	101 ± 18
Stomach	190 ± 50	127 ± 18	136 ± 24	133 ± 28	114 ± 8
Intestine	2130 ± 120	1750 ± 380	1830 ± 250	1760 ± 230	1630 ± 270
Kidneys	297 ± 23	258 ± 12	281 ± 20	280 ± 18	240 ± 30
Liver	1140 ± 50	1010 ± 50	1080 ± 70	1040 ± 60	930 ± 80
Heart	102 ± 4	89 ± 9	88 ± 11	91 ± 3	77 ± 4
Lung	170 ± 30	125 ± 5	133 ± 12	135 ± 11	150 ± 50
Muscle	360 ± 50	330 ± 140	350 ± 50	250 ± 40	230 ± 110
Femur	38 ± 10	31 ± 5	38 ± 2	35 ± 7	24 ± 7
Carcass	15,480 ± 450	13,600 ± 1500	14,400 ± 1200	13,200 ± 1100	11,500 ± 1800
MDA-MB-231 tumour	770 ± 260	610 ± 350	1060 ± 370	560 ± 180	790 ± 210
SKOV-3 tumour	240 ± 110	640 ± 690	430 ± 470	940 ± 440	770 ± 660

In case of HER2+ tumours (SKOV-3) an increasing uptake between 1 h p.i. and 1 day p.i. can be seen up to average 50 ± 14% ID/g. On the other hand, no major uptake was found in HER2- tumour (MDA-MB-231) — average value of 5.0 ± 1.7% ID/g. Excluding spleen, the uptake in HER2+ tumour has always been higher than in case of non-target tissues and HER2- tumour. The tumour-to-tissue ratio on day 7 p.i. for liver was 8, for kidneys 13 and for blood up to 20.

## Discussion

HER2+ tumours belong to aggressive subtypes of oncological diseases with poor response to chemotherapy. Immunotherapy is thus usually the preferred treatment option. In the case of HER2+ breast cancer, monoclonal antibodies, such as trastuzumab and pertuzumab are used. Both mentioned antibodies have already demonstrated that they can also be used as targeting vectors for radioimmunodiagnostics and therapy, due to their biggest advantage of known mechanism of bonding and interaction with HER2 receptors including internalization abilities which significantly help to minimize the impact of the recoil effect, which is observed in particular in case of α emitters (Larson et al. 2015; Mitri et al. 2012; Iqbal and Iqbal 2014; Hooijman et al. 2024).

When considering the choice of radionuclide to be paired with the monoclonal antibody for means of radioimmunotherapy, *β*^−^ emitters plays a significant role as they are still the most used therapeutic radionuclides in nuclear medicine. Nevertheless, *α* emitters have emerged as a promising alternative due to α particle properties, such as higher LET and lower range in tissue, resulting in enhanced effectiveness. Actinium-225 is often the *α* emitter of choice due to its chemical and physical qualities (Morgenstern et al. 2018; Hooijman et al. 2024).

In this study, conjugates of DOTA chelator and HER2 targeting antibodies, trastuzumab and pertuzumab, were obtained and optimal coupling conditions were found. The synthetic approach used creation of a peptide bond between carboxylic group of activated DOTA chelator (DOTA-NHS) and *ε*-amino group of lysine residues in the protein chain of antibodies. The other frequent option would be the use of *p*-SCN-Bn-DOTA bifunctional chelator when a thiourea bond between chelator and *ε*-amino group of lysine residues is formed (Zeglis and Lewis 2011). However, in (Maguire et al. 2014) it was found out that conjugates of HuM195 antibody synthesized using either DOTA-NHS or *p*-SCN-Bn-DOTA labelled with ^225^Ac show comparable stability in human serum even though *p*-SCN-Bn-DOTA provides one additional oxygen donor. DOTA-NHS was then the bifunctional chelator of choice as it enables the use of UV–Vis spectrometry for determination of conjugate concentration without further corrections.

It was found out that in order to obtain the same amount of conjugated DOTA molecules for both antibodies, in case of pertuzumab double the amount of DOTA-NHS bifunctional chelator is needed for reaction. This difference in the result of conjugation of these two antibodies has not been described, yet. Given the fact that both antibodies belong to IgG1 group of monoclonal antibodies with similar molar weights and similar number of lysine residues in the structure, the critical parameter would be higher structures of antibodies and a slightly different number of available lysine residues.

In the case of DOTA-pertuzumab conjugate, two samples with different numbers of conjugated DOTA molecules were prepared. In radiolabelling studies, it was confirmed that better radiolabelling results are achieved with the sample containing more DOTA molecules (PD2), which is a logical outcome. In order to achieve the highest possible radiolabelling yields and specific activity, it would seem logical to maximize conjugated chelator molecules. However, every other chelator molecule leads to change in molar weight and can cause significant changes in physical and chemical properties which can change the immunoreactivity, pharmacokinetics and biodistribution of conjugates (Karczmarczyk et al. 2022). A conservative estimate of the number of conjugated chelator molecules without any impact on antibody properties is 1–2 (Chomat et al. 2021). However, such a low number could not be sufficient for relevant radiolabelling yields. Most studies of radiolabelling HER2 targeting monoclonal antibodies use conjugates with 3–6 DOTA molecules (Abbas et al. 2011; Rasaneh et al. 2010a, b; Menon et al. 2022). This study also confirms the sufficiency of 6 DOTA molecules per one molecule of antibody for both tested antibodies.

For radiolabelling with ^225^Ac a one-step procedure was used. DOTA chelator usually requires elevated temperature for sufficient radiolabelling yields. It is a limiting factor for conjugates with antibodies. Consequently, a two-step procedure was previously used, implementing radiolabelling of DOTA prior to the conjugation. That of course lead to low yields around 6–17% (Borchardt et al. 2003; McDevitt et al. 2002). However, one-step procedure with elevated temperature up to 37 °C and prolongation of radiolabelling time to 1 h leads to reasonable yields, which are up to 10 times higher than in case of the two-step method, and 30-times higher specific activity (Thiele and Wilson 2018; Maguire et al. 2014). Due to ^225^Ac half-life of 9.92 days, this procedure has proven itself to be feasible also in this study where yields above 75% were reached.

In order to maximize specific activity and radiolabelling yields, an optimization study was performed. For both radioconjugates, the optimal radionuclide:conjugate ratio was determined to be 1:2000, which corresponds to approximately 1:12,000 for the radionuclide:chelator ratio. In the case of DOTA-pertuzumab the maximal radiolabelling yield was achieved after 90 min. Conversely, the radiolabelling kinetics of DOTA-trastuzumab was slower. In order to standardize the radiolabelling procedure, period of 120 min was chosen as an optimal radiolabelling time for both conjugates. The same period was used in (Maguire et al. 2014) for ^225^Ac immunoconjugate radiolabelling. The specific activity of 1.21 MBq/mg for trastuzumab and 1.26 MBq/mg for pertuzumab is in good agreement with ^227^Th-DOTA-trastuzumab with specific activity 1.0–1.6 MBq/mg (Abbas et al. 2011) or ^225^Ac-DOTA-trastuzumab with specific activity 1.85–5.55 MBq/mg in (Kondo et al. 2023).

The stability of radiolabelled compounds with prospect for use in nuclear medicine is a crucial property in general. In case of *α* emitter, the need for high stability of prepared radioconjugate is particularly more demanded due to higher destructive potential of these radionuclides. Both tested radioconjugates, ^225^Ac-TD1 and ^225^Ac-PD2, provided high stability in both PBS and FBS at room temperature for 10 days with radiochemical purity over 90% (see Fig. 4). Such a high stability and the known internalization mechanism of both antibodies should ensure the minimization of the recoil effect impact on the organism.

In other studies with ^225^Ac-labelled conjugates, the same level of stability was achieved. In (Pruszynski et al. 2018) radioconjugate ^225^Ac-DOTA-2Rs15d provided radiochemical purity above 90% after 10 days of study and the difference between the radiochemical purity of sample stored in PBS and human serum albumin was 3 percentage points. In a separate study of (Maguire et al. 2014), the radiochemical purity of DOTA-HuM195 conjugates radiolabelled with ^225^Ac was around 95–97% for 25 days. Similar data were gained for DOTA-HuM195 radiolabelled with either ^225^Ac or ^177^Lu stored in human serum for 15 days in (McDevitt et al. 2001). On the other hand, in (Rasaneh et al. 2010a) the radiochemical purity of conjugate ^177^Lu-DOTA-trastuzumab stored under similar conditions decreased to 81%.

Both conjugates, ^225^Ac-TD1 and ^225^Ac-PD2, exhibited high specificity towards HER2 receptors on SKOV-3 cells, while minimal binding was observed on the low HER2 expressing cell line MDA-MB-231. Moreover, the binding of both conjugates can be blocked or reduced by the excess of each cold antibody, likely due to the size of the antibody. Even though trastuzumab binds to domain IV of the HER2 receptor, the size of its molecule causes steric hindrances and prevent pertuzumab from binding to domain II of this receptor and vice versa.

The conjugate ^225^Ac-PD2 showed a higher binding affinity towards HER2 receptor as its dissociation constant *K*_D_ was determined to be 2.4 ± 0.2 nM comparing to 9 ± 3 nM measured for ^225^Ac-TD1. This finding is consistent with reported studies of unlabelled monoclonal antibodies (Cruz et al. 2023; Lua et al. 2018) as well as radiolabelled conjugates. In (Kondo et al. 2023) *K*_D_ of ^111^In-DOTA-trastuzumab was 12 ± 3 nM and in (Guleria et al. 2020) *K*_D_ of ^177^Lu-DOTA-trastuzumab was 13.61 nM. On the other hand, *K*_D_ of ^89^Zr-DFO-pertuzumab was 2.2 ± 0.4 nM in (Kang et al. 2022).

For biodistribution study, the highest safe activity of ^225^Ac-PD2 for administration was determined through a literature review to be 10 kBq, based on the biodistribution rate of antibodies and the radiation toxicity of ^225^Ac (Scheinberg and McDevitt 2011). However, given the low activities, the imaging of studied subjects using SPECT modality was not feasible.

In ex vivo biodistribution study of ^225^Ac-PD2 in normal mice, higher uptake was observed in highly blood perfused organs as it was difficult to get the tissue rid of the blood completely. In case of renal uptake, also other factors may have contributed to higher uptake values (up to 10% for both normal and tumour-xenografted mice). It is most probably caused by elimination of radioconjugate and possible renal excretion of ^225^Ac-DOTA (Deal et al. 1999). However, the tubular reabsorption of antibody metabolites can also contribute to higher renal retention of activity and radiation burden. Nephrotoxicity is a common problem observed for radiopharmaceuticals and can be dealt with by multiple approached. The most common way is the pre- or co-administration of compounds preventing the reuptake by blocking the endocytic receptors in the proximal tubule such as amino acids or gelatine-based plasma expanders (Chigoho et al. 2021; de Roode et al. 2024). The best example would be the administration of amino acids prior to therapy with ^177^Lu-DOTA-TATE (Lutathera®) (Hennrich and Kopka 2019).

The elevated uptake observed in the liver results from the metabolism of the radioconjugate. The conjugate is also metabolised in the spleen, however, such a high uptake with an increasing character on day 7 p.i. was surprising. Together with this finding, the gradually increasing spleen atrophy caused probably by ionizing radiation was noticed. The average weight of this organ indeed decreased from 81 ± 8 mg at 1 h p.i. to 20 ± 7 mg at 7 days p.i., resulting in a significant increase in uptake expressed in % ID/g. However, the activity accumulated in the spleen remained at an average of 0.65 ± 0.11% ID throughout the study.

In the case of tumour-xenografted mice, even higher values of uptake in the spleen were observed (average value of 37 ± 20% ID/g), although also standard deviation (SD) is noticed, resulting from the observed variation in spleen size (see Table 6). It is probably due to two conflicting influences. The influence of ionizing radiation causing the atrophy of the spleen as confirmed in the study with normal mice, and the influence of tumour-spleen interaction. Many works studied the mutual influence of spleen as an important immunity organ and tumorigenesis (Gabrilovich et al. 2012; Ugel et al. 2012; Bronte and Pittet 2013; Beheshti et al. 2015; Xiao et al. 2022). This influence is very often accompanied by splenomegaly in cancer cell transplantable models and positive correlations between spleen and tumour size were observed (Talmadge and Gabrilovich 2015; Hodgson et al. 2016; Mertens et al. 2018).

As a results of that, the tumour-spleen interaction and its impact on biodistribution is difficult to be evaluated in case of our study as two cancer cell lines, which can have a different effect on the host, were used for one testing subject. Moreover, the variable of radiation burden which differs between testing groups comes to the equation. Under such conditions no correlation analysis and no universal conclusion for spleen-tumour interaction can be taken.

Nevertheless, this unexplained phenomenon most certainly plays a role in a non-evident atrophy of spleen. According to gathered data there is no decrease in spleen size during the observed period from 1 h to 7 days p.i. (*P* > 0.5). However, the SD in spleen size is relatively high, thus hindering the ability to make assumptions about the level of atrophy.

The observed discrepancy in the uptake in spleen observed for normal and tumour-xenografted mice may be explained by mouse strain employed. Immunodeficient strains lacking B-lymphocytes, such as SCID, NOG or NRG, exhibit higher uptake in spleen than strains with B-lymphocytes, such as BABL/c, Swiss, athymic nude or Nu/Nu (Kang et al. 2022; Kondo et al. 2023; Sharma et al. 2018; Guleria et al. 2020; Menon et al. 2022).

In (Guleria et al. 2020), the biodistribution of ^177^Lu-DOTA-trastuzumab was tested in Swiss mice and the uptake in spleen was comparable with other non-accumulating organs. In work of (Borchardt et al. 2003) the uptake of ^111^In-DOTA-trastuzumab in spleen of tumour-xenografted athymic nude mice was around 15% at 24 h p.i. On the other hand, the same conjugate showed uptake of almost 40% ID/g in spleen when tested in HER2 + tumour-xenografted NRG mice. In (Marquez et al. 2014), the radioconjugate ^89^Zr-DOTA-pertuzumab tested on BT-474 (HER2 +) and MDA-MB-231 (HER2-) tumour-xenografted NOG mice showed the uptake in spleen over 100% ID/g, even though the uptake in Nu/Nu strain was under 3% ID/g. Similar results were gained in (Sharma et al. 2018) with ^89^Zr-DOTA-trastuzumab tested in NSG and Nu/Nu mouse strain. The uptake in spleen for NSG mice was up to 300% and the uptake in spleen for Nu/Nu mice was 6% ID/g. The authors of the paper expressed a theory of interaction between Fc domain of the antibody and Fc receptor of myeloid cells. After administration of isotype monoclonal antibody excess the uptake of radioconjugate in the spleen decreased 10 times as the Fc mediated non-specific uptake was reduced. In the light of above-mentioned data, the uptake in spleen would be a source of major discussion during potential transfer to clinical studies.

When transferring from normal to tumour-xenografted mice, no significant change in uptake of ^225^Ac-PD2 in other organs was observed except for stable average uptake of 4.3 ± 1.5% ID/g in bones comparing to slightly increasing uptake (from 5.9 ± 1.2% ID/g to 8.4 ± 1.3% ID/g, between 3 and 7 days p.i.) in the case of normal mice. The uptake in bones is generally caused by free ^225^Ac (Pruszynski et al. 2018; Kondo et al. 2023). Such low levels of activity uptake in bones then confirms the stability of radioconjugate in vivo suggested by in vitro tests. The differences in uptake pattern for immunocompetent (BALB/c) and immunodeficient (SCID) mice could indicate partial uptake by bone marrow. However, the separation of bone marrow would be necessary to confirm this hypothesis.

The most significant parameter of ex vivo biodistribution study is the level of uptake in tumour. In the case of this study, the xenografted mice carried both HER2 overexpressing tumour SKOV-3 and low HER2 expressing tumour MDA-MB-231. For SKOV-3 tumour the uptake was consistently high (around 50% ID/g) from 1st to 7th day p.i. In contrast, the accumulation in MDA-MB-231 tumour was comparable with healthy tissues (below 5% ID/g) for the whole period of the study. That confirms the results from in vitro stability study and the cell studies, which demonstrated a high binding affinity to HER2 receptors.

The comparison of tumour uptake with other studies is not straightforward due to the involvement of many parameters, such as mouse strain, tumour cell line or applied activity. However, a brief overview of the published results can be given. In (Menon et al. 2022) the conjugate ^177^Lu-DOTA-pertuzumab was tested in SCID-Beige mice with SKOV-3 and SK-BR-3 tumours (both HER2 overexpressing). In SKOV-3 tumour the increasing uptake was observed reaching 25% ID/g after 5 days post injection. Similar results were gained for the other tumour. The diagnostics radioconjugate ^89^Zr-DOTA-pertuzumab tested in (Kang et al. 2022) exhibited elevated uptake in HER2 + tumour (JIMT-1 cell line) of about 18% ID/g after 7 days p.i., and minimal accumulation in HER2- tumour (MDA-MB-231) with less than 8% ID/g. The same radioconjugate was tested in NOG mice bearing BT-474 (HER2 +) and MDA-MB-231 tumours, reaching the uptake of around 47% ID/g in HER2 + tumour with high SD of 32% ID/g and around 9.5% ID/g in HER2- tumour (Kang et al. 2022).

If we would like to compare the results of an ex vivo biodistribution study with the same radionuclide, ^225^Ac, it is necessary to transition from pertuzumab to trastuzumab, given that no study of ^225^Ac-DOTA-pertuzumab was carried out in the past. In (Borchardt et al 2003), the conjugate ^225^Ac-DOTA-trastuzumab visualised via ^111^In-DOTA-trastuzumab demonstrated the uptake in SKOV-3 tumour in the athymic nude mouse strain of about 26% ID/g at 1 day after i.v. application. After i.p. application the uptake in the tumour rose to 65% ID/g. The same radioconjugate reached in (Kondo et al. 2023) the uptake of 10% in HER2 + tumour in NRG mice at 48 h p.i.

In comparison with the majority of the studies investigating the biodistribution of radiolabelled HER2 targeting monoclonal antibodies, the radioconjugate ^225^Ac-PD2 demonstrates high uptake in HER2+ tumour and similarly low uptake in both HER2- tumour and healthy tissues with spleen uptake being the most variable.

## Conclusion

In the present study, two perspective radioconjugates of HER2 targeting monoclonal antibodies, ^225^Ac-DOTA-trastuzumab and ^225^Ac-DOTA-pertuzumab, were prepared. The preparation and testing of ^225^Ac-DOTA-pertuzumab was described for the very first time. Both conjugates demonstrated excellent stability. The biological activity of the antibodies was not severely compromised either by number of conjugated chelator molecules or the introduction of α emitter into the structure. In vitro studies with the prepared radioconjugates confirmed excellent stability and high HER2 binding specificity. An ex vivo biodistribution study of new radioconjugate ^225^Ac-DOTA-pertuzumab in normal and tumour-xenografted mice showed high uptake in HER2+ tumour over HER2- tumour and healthy tissues. These finding support the results of in vitro studies and demonstrate high potential of the compound for further studies.

## Supplementary Information

Supplementary meterials 1.

## Data Availability

Most raw data are listed in the article or supplementary information. Other raw data are available from the corresponding author upon a reasonable request.

## Abbreviations

ADC: Antibody-drug conjugate
DOTA: 1,4,7,10-Tetraazacyclododecane-1,4,7,10-tetraacetic acid
DOTA-NHS: 1,4,7,10-Tetraazacyclododecane-1,4,7,10-tetraacetic acid mono-*N*-hydroxysuccinimide ester
FBS: Fetal bovine serum
FDA: Food and drug administration
HER2: Human epidermal growth factor receptor type 2
i.p.: Intraperitoneal
i.v: Intravenous
LET: Linear energy transfer
MALDI-TOF-MS: Matrix-assisted laser desorption/ionization time of flight mass spectrometry
PBS: Phosphate buffered saline
PET: Positron emission tomography
p.i.: Post injection
RT: Room temperature
SCID: Severe combined immunodeficiency
SD: Standard deviation
SPECT: Single photon emission computed tomography
TAT: Targeted alpha therapy
