# Supplementary material for: In vitro and in vivo evaluation of anti-HER2 antibody conjugates labelled with 225Ac
Source: EJNMMI Radiopharm Chem. 2025 Apr 4;10:16. doi: 10.1186/s41181-025-00337-8 (PMC11971111; doi:10.1186/s41181-025-00337-8)
Supplement: Supplementary file 1 — Supplementary meterials 1. [file 41181_2025_337_MOESM1_ESM.pdf]

In vitro and *in vivo* evaluation of anti-HER2 antibody conjugates labelled with ^{225}Ac

Kateřina Ondrák Fialová[1*], Lukáš Ondrák[1], Martin Vlk[1], Ján Kozempel[1], Kateřina Nováková[2], Zbyněk Nový[3], Katarína Hajduová[3], Marián Hajdúch[3], Miloš Petřík[3], Marek Pruszynski[4,5], Frank Bruchertseifer[6], Alfred Morgenstern[6]

[1]*Department of Nuclear Chemistry, Faculty of Nuclear Sciences and Physical Engineering, Czech Technical University in Prague, Břehová 87/7, 115 19 Prague, Czech Republic*

[2] *Institute of Organic Chemistry and Biochemistry of the CAS, Flemingovo nameští 542/2, 16000, Prague, Czech Republic*

[3]*Institute of Molecular and Translational Medicine, Faculty of Medicine and Dentistry Palacký University Olomouc, Hněvotínská 5, 779 00 Olomouc 9*

[4]*Institute of Nuclear Chemistry and Technology, Dorodna 16, 03-195 Warsaw, Poland*

[5]*NOMATEN Centre of Excellence, National Centre for Nuclear Research, Andrzeja Soltana 7, 05-400 Otwock, Poland*

[6]*European Commission, Joint Research Centre, Karlsruhe, Germany*

**corresponding author*

Electronic Supplementary Information (ESI)

Table of content

MALDI-TOF-MS spectra of prepared conjugates 2

Preliminary radiolabelling of prepared conjugates 4

MALDI-TOF-MS spectra of prepared conjugates

The MALDI-TOF spectra were measured on UltrafleXtremeTM MALDI-TOF/TOF mass spectrometer (Bruker Daltonics, Germany) with 1 kHz smartbeam II laser. The measurements were done in positive linear mode technique, with the mass range 20-300 kDa. The accelerating voltage was set at 25kV. Typically, spectra were obtained by accumulating 25000 shots. In Fig. S1-S5 there are spectra of non-conjugated antibodies and prepared conjugates.

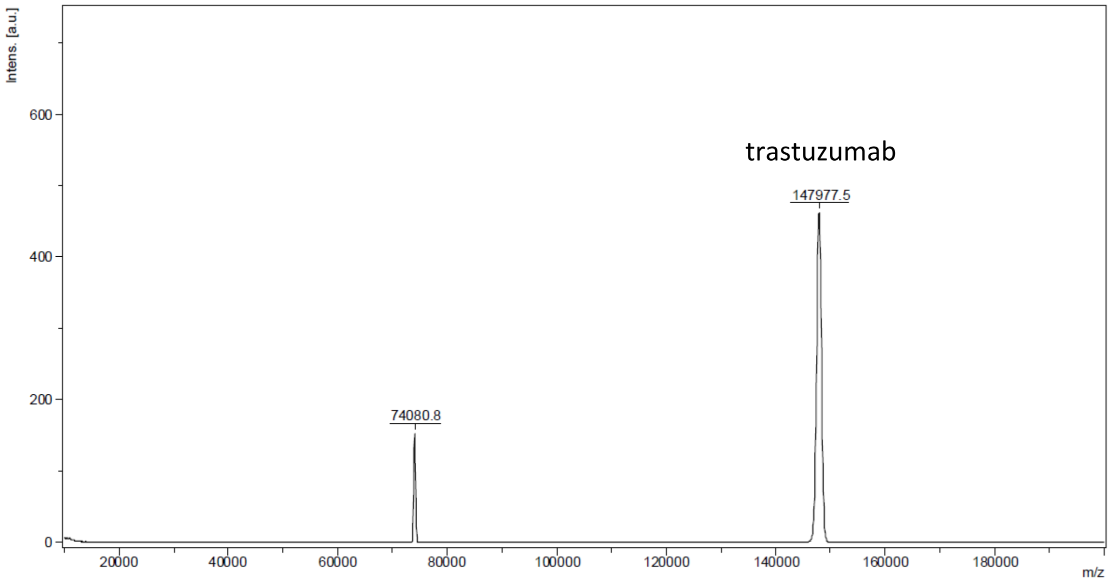

Figure S1 MALDI-TOF-MS spectrum of trastuzumab

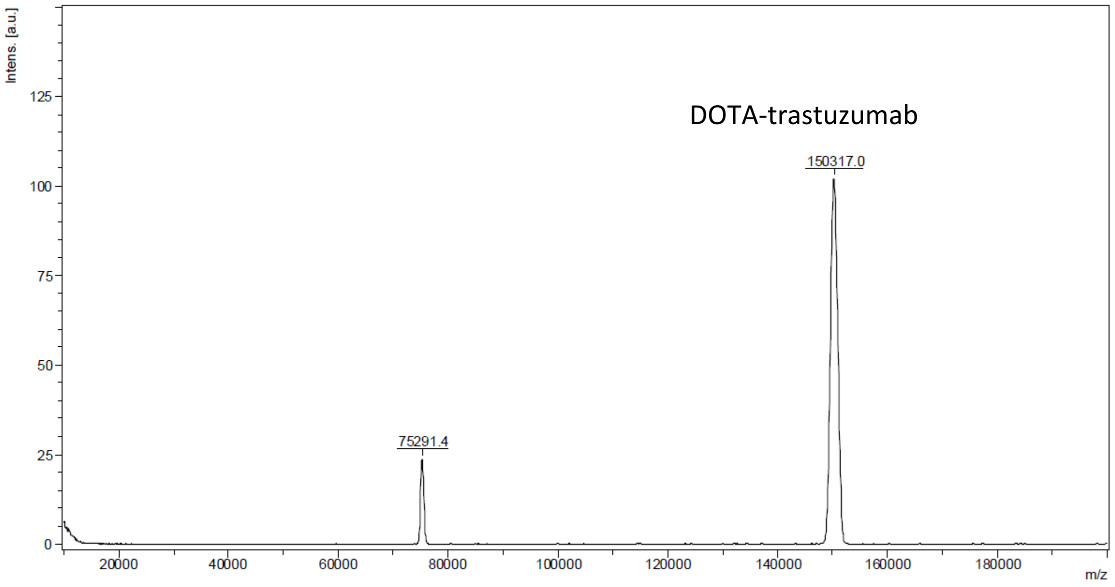

Figure S2 MALDI-TOF-MS spectrum of conjugate DOTA-trastuzumab (TD1)

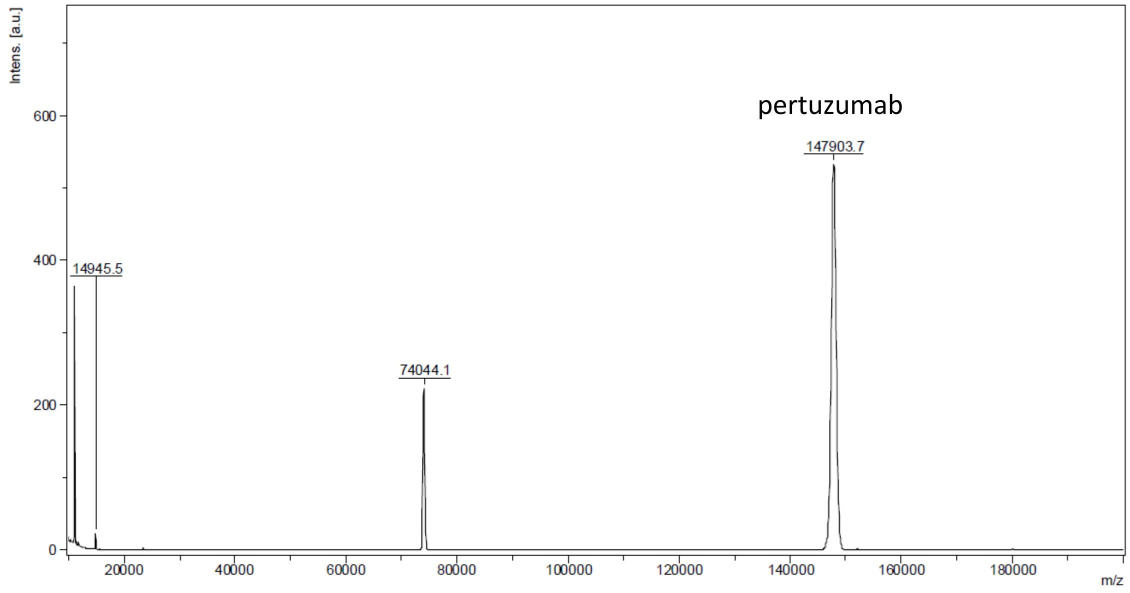

Figure S3 MALDI-TOF-MS spectrum of pertuzumab

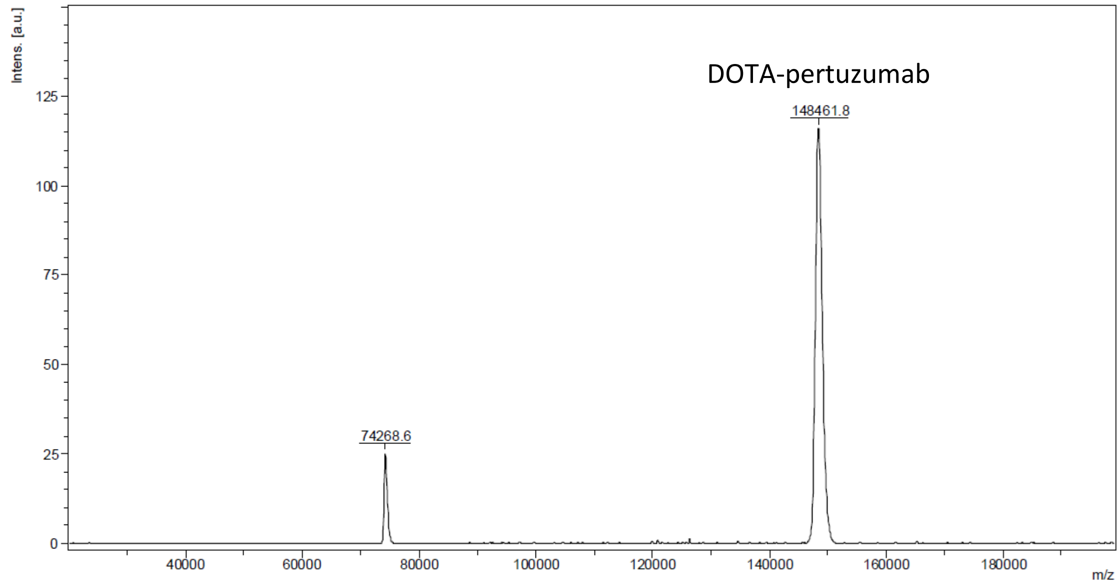

Figure S4 MALDI-TOF-MS spectrum of conjugate DOTA-pertuzumab (PD1)

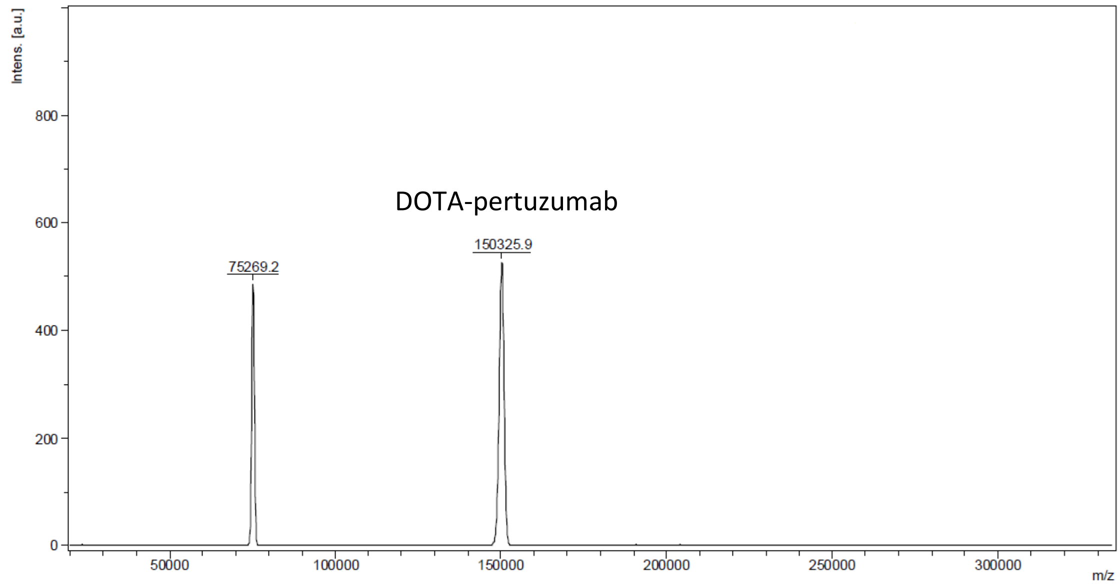

Figure S5 MALDI-TOF-MS spectrum of conjugate DOTA-pertuzumab (PD2)

Preliminary radiolabelling of prepared conjugates

The prepared conjugates were radiolabelled with ^{225}Ac in various radionuclide:conjugate ratios in 0.02 M ammonium acetate (pH 6) at 37 °C for 2 h in reaction volume 200 µl. Radiolabelling yields were determined by thin layer chromatography (TLC) on ITLC-SG (Agilent Technologies, USA) paper-strips using 0.05 M natrium citrate (pH 5.5). In this arrangement the radiolabelled conjugate was retained at the origin (R_f = 0.0), free ^{225}Ac moved with the front (R_f = 0.8-1.0) and the complex of free DOTA with ^{225}Ac remained at R_f = 0.6-0.8. The radiochromatograms were measured on TLC scanner AR 2000 (Bioscan Inc., USA) after 24 h. The model radiochromatograms for radionuclide:conjugate ratio 1:2000 at t = 0 min and 120 min for TD1, PD1 and PD2 are in Fig. S6-S8, respectively.

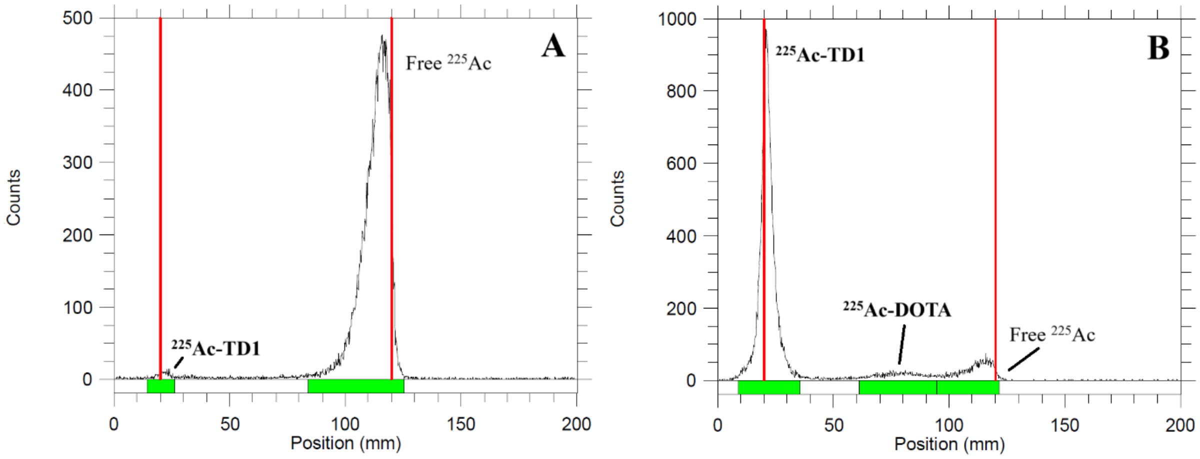

Figure S6 Radiochromatograms of ^{225}Ac-TD1: ratio 1:2000: (A) t = 0 min, (B) t = 120 min

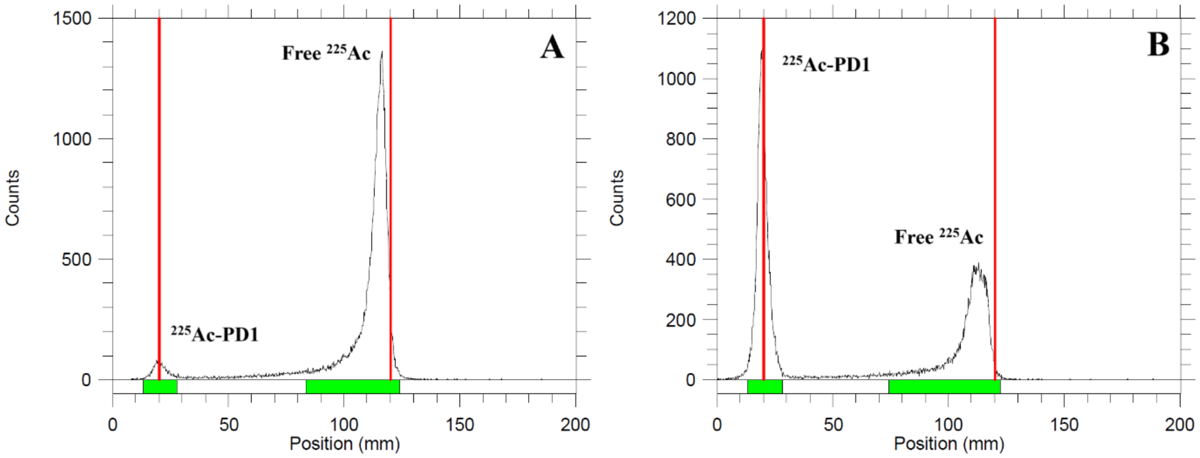

Figure S7 Radiochromatograms of ^{225}Ac-PD1: ratio 1:2000: (A) t = 0 min, (B) t = 120 min

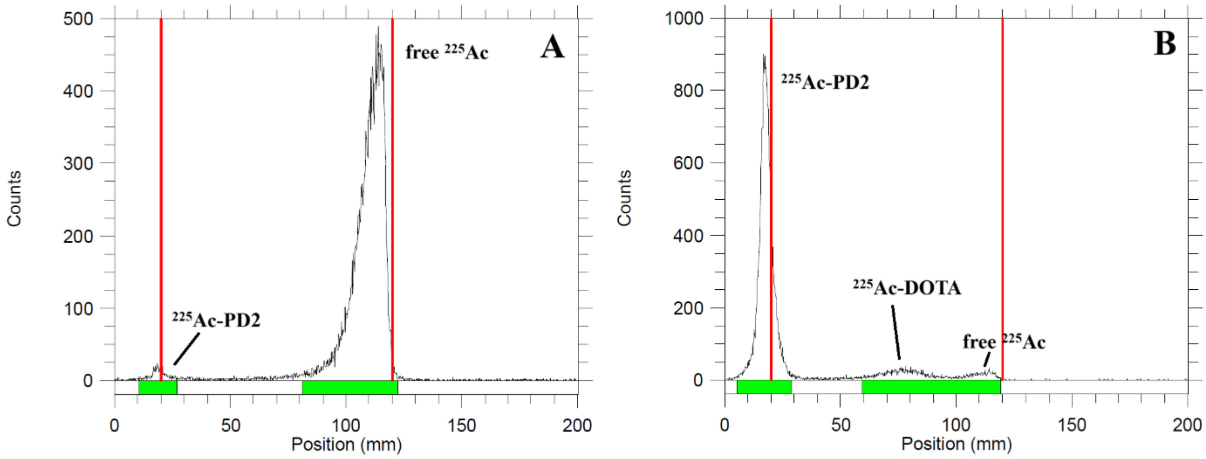

Figure S8 Radiochromatograms of ^{225}Ac-PD2: ratio 1:2000: (A) t = 0 min, (B) t = 120 min
